# Enteropathy Markers in Early Life Were Associated with Adipokine, Apolipoprotein, and Cytokine Profiles Consistent with an Adverse Cardiometabolic Disease Risk Profile Later in Childhood in a Peruvian Birth Cohort

**DOI:** 10.4269/ajtmh.21-1024

**Published:** 2022-09-12

**Authors:** Josh M. Colston, Yen Ting Chen, Patrick Hinson, Nhat-Lan H. Nguyen, Pablo Peñataro Yori, Maribel Paredes Olortegui, Dixner Rengifo Trigoso, Mery Siguas Salas, Richard L. Guerrant, Ruthly François, Margaret N. Kosek

**Affiliations:** ^1^ Division of Infectious Diseases and International Health, University of Virginia School of Medicine, Charlottesville, Virginia;; ^2^ Department of Emergency Medicine, Chi-Mei Medical Center, Tainan, Taiwan;; ^3^ College of Arts and Sciences, University of Virginia, Charlottesville, Virginia;; ^4^ Asociación Benéfica Prisma, Unidad de Investigaciones Biomédicas, Iquitos, Peru;; ^5^ Center for Global Health Equity, University of Virginia School of Medicine, Charlottesville, Virginia;; ^6^ School of Medicine, University of North Carolina at Chapel Hill, Chapel Hill, North Carolina;; ^7^ Division of Public Health Sciences, University of Virginia School of Medicine, Charlottesville, Virginia

## Abstract

Metabolic syndrome is a cluster of risk factors for cardiovascular disease afflicting more than 1 billion people worldwide and is increasingly being identified in younger age groups and in socioeconomically disadvantaged settings in the global south. Enteropathogen exposure and environmental enteropathy in infancy may contribute to metabolic syndrome by disrupting the metabolic profile in a way that is detectable in cardiometabolic markers later in childhood. A total of 217 subjects previously enrolled in a birth cohort in Amazonian Peru were monitored annually from ages 2 to 5 years. A total of 197 blood samples collected in later childhood were analyzed for 37 cardiometabolic biomarkers, including adipokines, apolipoproteins, cytokines, which were matched to extant early-life markers of enteropathy ascertained between birth and 2 years. Multivariate and multivariable regression models were fitted to test for associations, adjusting for confounders. Fecal and urinary markers of intestinal permeability and inflammation (myeloperoxidase, lactulose, and mannitol) measured in infancy were associated with later serum concentrations of soluble CD40-ligand, a proinflammatory cytokine correlated with adverse metabolic outcomes. Fecal myeloperoxidase was also associated with later levels of omentin-1. Enteric protozoa exposure showed stronger associations with later cardiometabolic markers than viruses, bacteria, and overall diarrheal episodes. Early-life enteropathy markers were associated with altered adipokine, apolipoprotein, and cytokine profiles later in childhood consistent with an adverse cardiometabolic disease risk profile in this cohort. Markers of intestinal permeability and inflammation measured in urine (lactulose, mannitol) and stool (myeloperoxidase, protozoal infections) during infancy may predict metabolic syndrome in adulthood.

## INTRODUCTION

Metabolic syndrome (MetS) refers to a cluster of risk factors for cardiovascular disease (CVD) including central obesity, hyperglycemia, hypertriglyceridemia, decreased high-density lipoprotein, and hypertension, which has long been recognized in adults and has more recently become the subject of research in children and adolescents.^1^ The syndrome is thought to afflict more than 1 billion people worldwide—three times the prevalence of diabetes—and is increasingly being identified in younger age groups, non-obese individuals and socioeconomically disadvantaged populations in the global south, once thought to be protected from such conditions because of the absence of “western” lifestyle and dietary patterns.^2^^–^^4^ MetS confers a 2-fold increased 10-year risk of CVD and a 5-fold increased lifetime risk of developing type 2 diabetes mellitus,^5^ and there are complex, multidirectional mechanisms connecting each of these conditions.^6^ Although MetS and its sequalae can be managed and prevented through dietary, behavioral, and lifestyle modifications, early identification and intervention are critical to avoid severe morbidities and the need for pharmaceutical or surgical intervention.^7^

Evidence has increased during the past several decades in support of the “Barker hypothesis” that many chronic and noncommunicable conditions of adulthood have developmental origins—they are “programmed” by exposures encountered in childhood or even in utero^8^^–^^11^—and MetS may be no exception. A hypothesis is forming that the etiology of this syndrome stretches into early infancy, encompassing risk factors as diverse as diarrhea,^10^ growth,^12^ systemic inflammation,^13^ and genetic and epigenetic factors.^2^^,^^14^ Such associations are challenging to characterize epidemiologically because they operate over the length of the life course; however, numerous insights have been gained through the follow-up of subjects at older ages who had previously been enrolled in birth cohort studies as infants.^10^^,^^15^ For example, DeBoer et al.^10^ identified a statistically significant 34% increase in the odds of MetS in adulthood for every 10% increase in diarrhea burden experienced during the first 6 months of life by monitoring participants in a longitudinal study of growth and development in Guatemala some 30 years later. Using similar study designs, undernutrition and suboptimal growth in early childhood have also been shown to be associated with a later life risk of CVD and glucose intolerance in both high- and lower-income countries.^12^ Elsewhere, gut barrier permeability has been demonstrated to have a role in MetS etiology,^16^ suggesting that intestinal injury from enteric infections, and specifically the process known as environmental enteropathy (EE), may explain the link with diarrhea and growth in early life.^13^ Distinct from Crohn’s disease, ulcerative colitis, or other chronic immune-regulated intestinal conditions, EE refers to the process by which successive, overlapping exposures to multiple enteric pathogens during infancy causes cumulative injury to the intestinal epithelium and, eventually, systemic inflammation and altered nutrient uptake and use.^17^

Because the pathogenesis of MetS and its associated sequalae take place over the life course as a result of cumulative exposures, there is an interest in identifying subclinical precursors that can be treated as biomarkers to classify individuals at elevated metabolic risk early in the disease process, to facilitate early effective interventions, and to avert significant morbidity and organ damage.^18^ This need is particularly salient given the competing diagnostic criteria for MetS, all of which rely on clinical features that occur late in the disease progression. Numerous analytes detectable in biologic samples are receiving attention in this regard, and several have had correlations established, usually concurrently, with clinical cardiometabolic outcomes. Concentrations of apolipoproteins, the transporters of lipids in the circulation, can be measured in the blood, and abnormal values for these markers may precede dyslipidemia and other components of MetS.^19^ Specifically, low concentrations of apolipoprotein (Apo)-AI, a component of high-density lipoprotein cholesterol, are associated with poor glucose metabolism and increased risk of CVD, whereas Apo-B is a marker of lower density lipoproteins that is more reliable than low-density lipoproteins, and increased levels are associated with cardiovascular risk.^19^^,^^20^ The adipokines leptin and adiponectin, and the ratio of the two, are promising markers of MetS. Proinflammatory cytokines including interleukin-6 (IL-6) and tumor necrosis factor-α (TNF-α) are directly associated with the risk of MetS, whereas anti-inflammatory cytokines—IL-10, ghrelin, and antioxidant factors (paraoxonase 1 [PON1])—are correlated inversely with components of the cluster.^18^^,^^21^^–^^24^ Several of these associations have been observed in school children and adolescents as well as adults,^25^^,^^26^ and mirror comparable associations with early-childhood growth observed in a Peruvian cohort and elsewhere.^27^^,^^28^ It is therefore possible to explore hypotheses about the early etiology of MetS using markers like these as surrogates, although there have been few published examples of this to date.^13^

The objective of this analysis was to identify and characterize associations between markers of infectious enteric disease exposure and enteropathy during the first 24 months of life, and adipokine, apolipoprotein, and cytokine profiles in later childhood in a Peruvian birth cohort. The hypothesis was that increased enteropathogen exposure and EE in infancy cause disruptions to the metabolic profile later in childhood that would be detectable in changes in the concentrations of relevant cardiometabolic markers.

## MATERIALS AND METHODS

### Conceptual framework.

**Figure 1.**
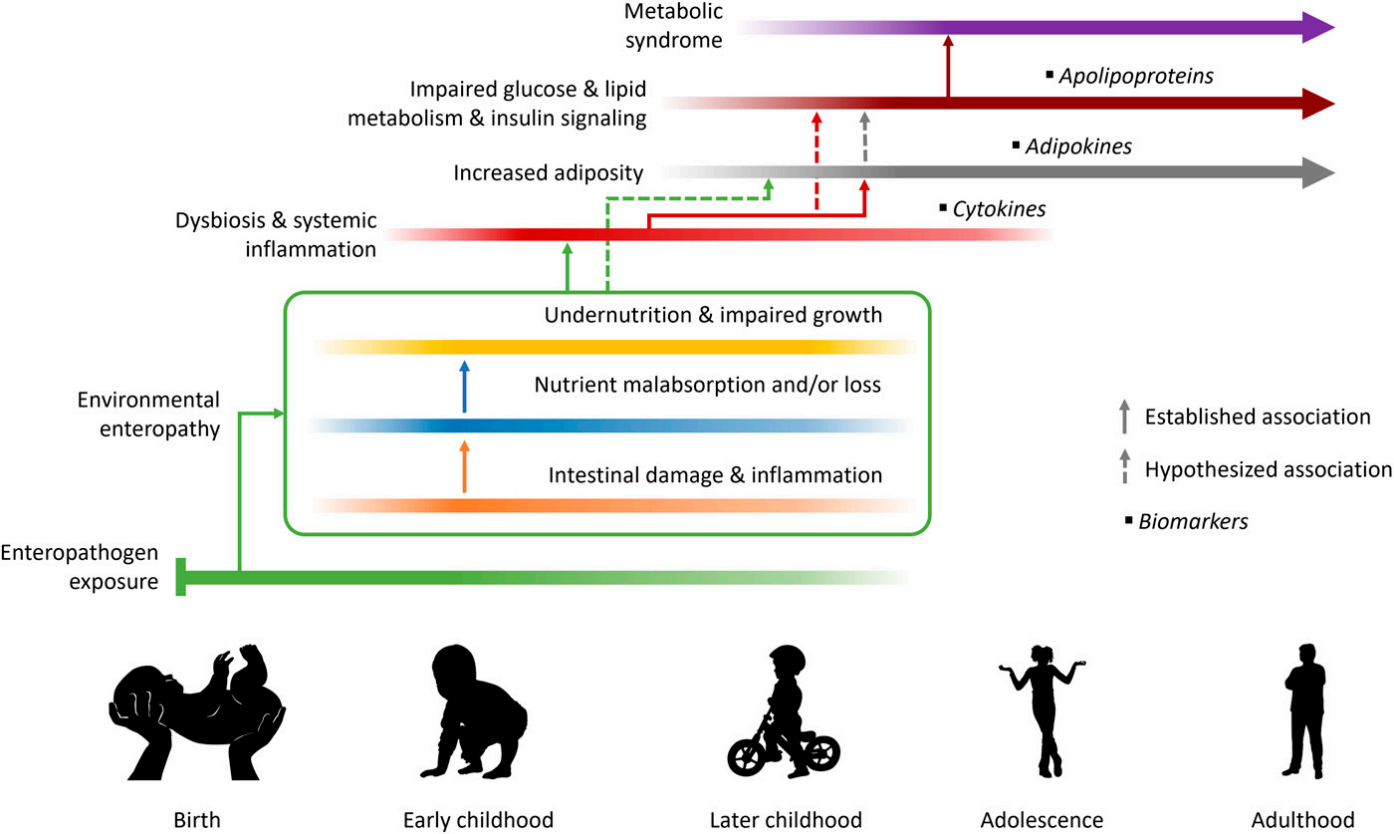
Established and hypothesized associations and causal pathways connecting early-life exposures with metabolic syndrome in adolescence and adulthood, and their biomarkers.

Figure 1 presents the established and hypothesized associations and causal pathways connecting early-life exposures with MetS in later childhood, adolescence, and adulthood that guided our analysis. For the purposes of this analysis, “early life” refers to the period from birth to 2 years, whereas “later childhood” refers to 2 years to adolescence. For children raised in conditions of poor sanitation, environmental exposure to enteropathogens starts early in infancy and leads to EE by disrupting normal intestinal function, altering the ultrastructure and function of the delicate and complex villi and microvilli.^29^ Cumulative damage to the gut’s surface increases its permeability to microbes and large molecules, provoking prolonged, low-level systemic inflammation (detectable in disruptions to cytokine profiles), and impairing uptake and altering the use of nutrients, which in turn lead to impaired growth and development.^27^ Over the course of later childhood, these processes increase adiposity and begin to impair irreversibly the body’s ability to metabolize glucose and lipids, and signal insulin, eventually increasing the risk of individuals developing MetS as they progress through adolescence to adulthood.^12^^,^^30^ In this analysis we tested the hypothesis that deviations from normal levels of biomarkers of inflammation, adiposity, and dyslipidemia that presage later MetS are detectable in childhood and can be identified through their associations with early-life enteropathy markers.

### Study population.

The Etiology Risk Factors and Interactions of Enteric Infections and Malnutrition and the Consequences for Child Health and Development (MAL-ED) project was a multi-site birth cohort study that ran from November 2009 to March 2014 and was established, in part, to investigate the impact of EE on early childhood growth and development.^31^ Subjects were recruited and enrolled from eight communities, each in a different low- and middle-income country according to criteria described previously^31^ (within 17 days of birth, ≥ 1,500-g birthweight, singleton birth, not ill, mother ≥ 16 years, family expecting to stay in community at least 6 months). Enrollees were monitored during their first 2 years of life through biweekly visits to generate a cumulative and continuous history of disease burden in early life. At the MAL-ED project site in Peru—the village of Santa Clara de Nanay in the exurbs of Iquitos, Loreto Province—contact was maintained with most of the original 303 subjects beyond the initial 2-year window through weekly visits until the children reached 5 years of age. Santa Clara de Nanay is situated in a low-lying tropical rainforest area at the confluence of several Amazon tributaries that is characterized by a lack of infrastructure, low access to adequate water and sanitation services, and a high burden of enteropathogen infection and undernutrition.^27^^,^^32^ The population of the study community is Spanish speaking and of mixed Hispanic and Amazonian Amerindian ethnicity. Some loss to follow-up occurred as the result of intermittent and permanent out-migration of this socioeconomically precarious population from the study site.

### Outcome variables.

Fasting blood samples were taken from subjects at 7 and 15 months of age, and then annually from ages 2 to 5 years. The most recently contributed sample from each of the 217 subjects with the most complete follow-up was identified and analyzed using multiplex Luminex magnetic bead panels for concentrations of a panel of 37 biomarker analytes including adipokines, apolipoproteins, and cytokines (Bio-Plex Pro^TM^ RBM Metabolic, Hercules, CA and Hormone and Human Th17 Cytokine Assays; Milliplex^®^ Human Metabolic Apolipoprotein and Hormone panels, Burlington, MA). In addition, the Apo B/A-I ratio was calculated and included as a marker. For most subjects (72% of those contributing any blood samples), a sample collected at the target age of 5 years was available for inclusion; otherwise, samples from the target ages of 2, 3, or 4 years (22%) were used. The minority of subjects (6%) for whom the most recent available sample was collected at 15 months were excluded from the analysis to avoid overlap in the periods of exposure (early life) and outcome ascertainment (later childhood). In addition to the serum outcome markers, the subjects’ last available body mass index (BMI)-for-age z-score value was included as a comparator clinical MetS outcome.^1^ All outcome variable units and distributions are summarized in Supplemental Table S1.

### Exposure variables.

The following primary exposure variables were taken from extant data collected from the subjects between birth and 2 years of age as part of the MAL-ED study.

#### Biomarkers of enteropathy.

The following biomarkers were included based on their hypothesized and documented associations with EE and growth outcomes in this cohort and elsewhere^27^^,^^29^^,^^33^: seven serum biomarkers (adiponectin [measured in micrograms per milliliter], ferritin [nanograms per milliliter], IL-8 [measured in picograms per milliliter], proline, serum amyloid P-component [micrograms per milliliter], serotransferrin [transferrin, milligrams per deciliter], and tryptophan [micromoles per liter]) measured in blood samples taken at 7, 15, and 24 months by previously described methods^27^; three fecal biomarkers (alpha-1-antitrypsin [measured in milligrams per gram], myeloperoxidase [MPO, measured in nanograms per milliliter], and neopterin [measured in nanomoles per liter]) measured in monthly stool samples by ELISA; the percentage of urinary lactulose and mannitol recovery as measured by intestinal permeability tests performed on urine samples collected at 3, 6, 9, 15, and 24 months after an oral lactulose and mannitol dosing as reported previously^34^; and the lactulose-to-mannitol ratio standardized as z-scores relative to a low-EE reference population.^35^

#### Nutritional status.

Length-for-age z-scores (LAZ) and weight-for-age z-scores (WAZ) calculated from results of monthly anthropometric assessments were included, as well as birthweight as a marker of nutritional status in utero.

#### Cumulative enteropathogen exposure.

Stool samples collected at monthly intervals and during caregiver-reported diarrheal episodes were tested for the presence of numerous enteropathogen species using quantitative polymerase chain reaction, ELISA, and microscopy methods.^36^^,^^37^ Episodes of infection with the pathogens with greatest attributable morbidity were summed over the 2-year follow-up period for three taxa: viruses (adenovirus, astrovirus, norovirus, rotavirus, and sapovirus), bacteria (*Aeromonas* spp., *Campylobacter* spp., *Salmonella* spp., *Shigella* spp., *Plesiomonas* spp., *Vibrio* spp., and various pathotypes of *Escherichia coli*), and protozoa (*Cryptosporidium* spp., *Entamoeba histolytica*, and *Giardia* spp.). Samples from the same subject that were positive for the same pathogen were considered discrete infection episodes if separated either by an intermediate negative sample or a period of 14 days, with the exception of *Campylobacter* spp. and norovirus, for which a period of 30 days was used, and the three protozoa for which three intermediate negative samples were required (criteria previously documented by Colston et al.^38^). The total number of diarrheal episodes was also included.

A minimal set of covariates was used in the analysis to adjust for potential confounding, including gender, average monthly household income, mother’s BMI at enrollment, and the proportion of days during the first 6 months of life in which the infant was breastfed exclusively (parental obesity and feeding practices being risk factors for early-onset MetS^1^). The definitions of all included variables are given in Table 1, and the distributions of the exposures and covariates are presented in Supplemental Table S2.

**Table 1:** Definitions of early-life enteropathy markers, later childhood metabolic syndrome markers that met the criteria for inclusion in the analysis, and covariates

Markers and covariates	Calculation
A. Early-life (0–2 years of age) enteropathy markers	
i. Serum markers	
Adiponectin, ferritin, interleukin-8, proline, serum amyloid P-component, transferrin, and tryptophan	Within-subject mean of concentrations measured at 7, 15, and 24 months of age
ii. Fecal markers	
Alpha-1-antitrypsin, myeloperoxidase, and neopterin	Within-subject mean of concentrations measured in monthly, non-diarrheal stools from ages 0 to 24 months
iii. Urinary markers	
Percent urinary lactulose and mannitol recovery	Within-subject mean of results measured at 3, 6, 9, 15, and 24 months of age
Z scores of lactulose-to-mannitol ratio relative to reference population	Within-subject mean of results measured at 3, 6, 9, and 15 months of age*
iv. Anthropometric markers	
Length- and weight-for-age z-scores	Within-subject means of monthly measurements from ages 0 to 24 months
Birth weight	Weight at birth
v. Enteropathogen exposure markers	
Viral, bacterial, protozoal infection, and diarrheal episodes	Within-subject total infections diagnosed in monthly and diarrheal stools from ages 0 to 24 months
B. Later childhood (2–5 years of age) MetS markers	
i. Apolipoproteins	
ApoA-I†, ApoA-II, ApoB, ApoB-to-ApoA-I ratio, ApoC-II, ApoC-III, ApoE	Concentration in most recently available blood sample
ii. Adipokines	
C-peptide, fibroblast growth factors 21 and 23, galectin-3, gastric inhibitory polypeptide, insulin, leptin, omentin-1†, pentraxin 3, paraoxonase 1†, pancreatic polypeptide†, soluble leptin receptor†, and visceral adipose tissue-derived serpin	Concentration in most recently available blood sample
iii. Cytokines	
Monocyte chemoattractant protein 1, soluble CD40-ligand, and tumor necrosis factor-α	Concentration in most recently available blood sample
iv. Clinical MetS marker	
Body mass index-for-age z-score	Calculated from most recent anthropometric assessment
C. Covariates	
i. Baseline factors	
Gender, average monthly household income	Ascertained at enrollment
ii. Age at most recent blood sample	Dichotomized at 48 months

Apo = apolipoprotein; MetS = metabolic syndrome.

* z-Scores of lactulose-to-mannitol ratio could not be calculated at 24 months because reference values were not available at that age.

† Biomarkers with documented inverse associations with MetS; all others have documented direct associations.

### Statistical analysis.

All biomarker values were log-transformed with base 2 so that each 1-U increase in effect size estimate was interpreted as that incurred by a doubling in biomarker concentration. Because the Luminex bead panel can only detect analyte concentrations within certain ranges of values that vary by analyte and subject age, many biomarkers had values that were greater than or, more commonly, less than the limits of detection for the assay; in some cases, these were a significant proportion of the overall data. Biomarkers for which more than 40% of the original values were missing were excluded from further analysis.^27^ For all other biomarkers, values that were outside the range of detection were imputed using interval regression, a method used for partially observed data with values that are missing because they were censored at known upper and lower limits, but are assumed to follow the distribution of the fully observed values.^39^ For the EE biomarkers and nutritional status exposures (apart from birthweight) the within-subject average value was calculated across all available observations in the birth to 2-year age window, whereas for the enteropathogen infection and diarrheal episodes, the total was used to approximate cumulative exposure. These extant summary exposure measures were matched to the later outcome biomarker concentrations from the same subjects.

Correlations between the MetS serum biomarker outcomes and the early-life exposures were plotted in a correlation matrix heat map. A multivariate regression model was then fitted to model associations jointly between the multiple early-life markers and each of the MetS biomarkers, adjusting for the covariates. To assess the statistical significance of each exposure’s association with the entire set of biomarker outcomes, and vice versa, *P* values from Wald tests of the combined significance of the relevant coefficient estimates were compared with a Bonferroni-corrected threshold of α = 0.05/*m*, where *m* is either the number of outcomes or the number of early-life markers, depending on which is being jointly tested. The BMI z-score comparator outcome was excluded from the multivariate model because of its strong association with the anthropometric exposures inflating their overall significance across the other outcomes, and was instead modeled as a single outcome. Last, a subset of early-life markers was selected based on the significance and strength of their associations across multiple outcomes and were included, with the same covariates, in a series of final regression models for each of the MetS biomarker outcomes. For the initial multivariate model, exposures were standardized so that their coefficients were on a similar scale and could be compared easily across multiple early-life–MetS marker pairings, whereas for the final multivariable models, they were kept on their original scale for ease of interpretation. The coefficients from the final models were visualized in a dot-and-whisker plot. Analyses were carried out using Stata 16 (StataCorp, College Station, TX) and R 3.6.2 (R Foundation for Statistical Computing, Vienna, Austria).

### Data statement.

At the time of publication, there were restrictions to the availability of the data used in the analysis. Readers who wish to request access to the data should contact the corresponding author.

## RESULTS

Samples from 217 subjects were analyzed for biomarkers, including adipokines, apolipoproteins, cytokines, hormones, peptides, proteins, and an enzyme, and 197 of these samples met the criterion for inclusion in the analysis. Summary statistics of the variables, including all candidate serum MetS biomarkers available for this analysis and their documented associations with individual MetS components based on a review of the published scientific literature, are presented in Supplemental Tables S1 and S2. Prior to applying the exclusion criteria, 37 distinct MetS biomarkers were initially considered for inclusion in further analysis. Fourteen analytes—amylin; ghrelin; glucagon-like peptide 1; glucagon; interferon-γ; IL-1β, -4, -6, -10, -17A, -22, -23, and -33; and peptide tyrosine tyrosine—were excluded from further analysis because more than 40% of their original values were missing (mostly because their concentrations were below the quantifiable range for the assay). The 23 analytes that met the inclusion criteria of retaining more than 60% of their original values included all of the apolipoproteins, the adipokines C-peptide, fibroblast growth factor (FGF) 21 and FGF23, galectin-3 (Gal-3), gastric inhibitory polypeptide (GIP), insulin, leptin, omentin-1, pentraxin 3, PON1, pancreatic polypeptide (PP), soluble leptin receptor (sOB-R), and visceral adipose tissue-derived serpin (Vaspin); and the cytokines monocyte chemoattractant protein 1 (MCP-1), soluble CD40-ligand (sCD40L), and tumor necrosis factor-α (TNF-α). Of these, five (omentin-1, ApoA-I, PON1, sOB-R, and PP) have a documented inverse association with MetS components overall, whereas the remaining 18 have direct associations reported previously in the literature (See citations in table S1). Table 1 shows the definitions and categories of the variables included in the analysis.

**Figure 2.**
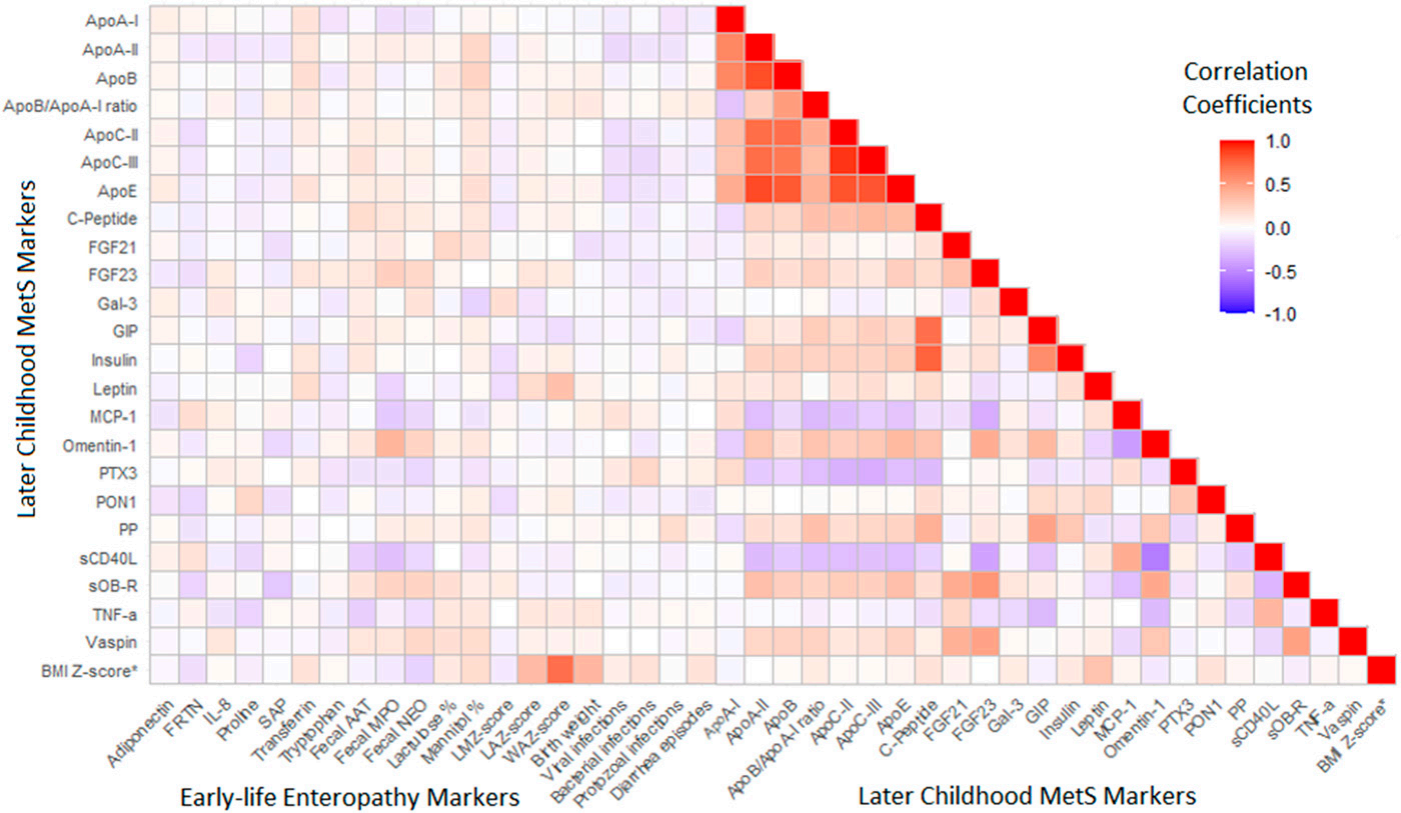
Heat map matrix of the correlations between each of the included later-childhood (2–5 years) serum metabolic syndrome (MetS) markers with each other and the early-life (0–2 years) enteropathy markers. AAT, alpha-1-antitrypsin; Apo, apolipoprotein; BMI, body mass index; FGF, fibroblast growth factor; FRTN, ferritin; Gal-3, galectin-3; GIP, gastric inhibitory polypeptide; IL, interleukin; LAZ, length-to-age z-score; LMZ, lactulose-to-mannitol ratio z-scores; MCP-1, monocyte chemoattractant protein 1; MPO, myeloperoxidase; NEO, neopterin; PON1, paraoxonase 1; PP, pancreatic polypeptide; PTX3, pentraxin 3; SAP, serum amyloid P-component; sCD40L, soluble CD40-ligand; sOB-R, soluble leptin receptor; TNF, tumor necrosis factor; Vaspin, visceral adipose tissue-derived serpin; WAZ, weight-to-age z-score. * BMI z-score is included as comparator clinical MetS outcome.

Figure 2 shows a heat map matrix of the Spearman coefficients for the correlations between each of the included serum MetS biomarkers with each other and with the early-life enteropathy markers. As expected, correlations between markers of early-life exposures with those measured later in childhood tended to be weaker than those between the later life biomarkers and each other. Notable exposure–outcome correlations include that of fecal MPO concentration, an acute intestinal inflammation marker, directly with omentin-1 and inversely with sCD40L, as well as WAZ with leptin. Among the correlations between MetS markers, the apolipoproteins demonstrated strong direct correlations exclusively with each other. Other strong correlations included C-peptide directly with both GIP and insulin, and the inverse correlation of omentin-1 with sCD40L. Later childhood BMI z-score correlated directly with the three early-life anthropometric exposures—most markedly with WAZ—and to a lesser extent with leptin.

**Figure 3.**
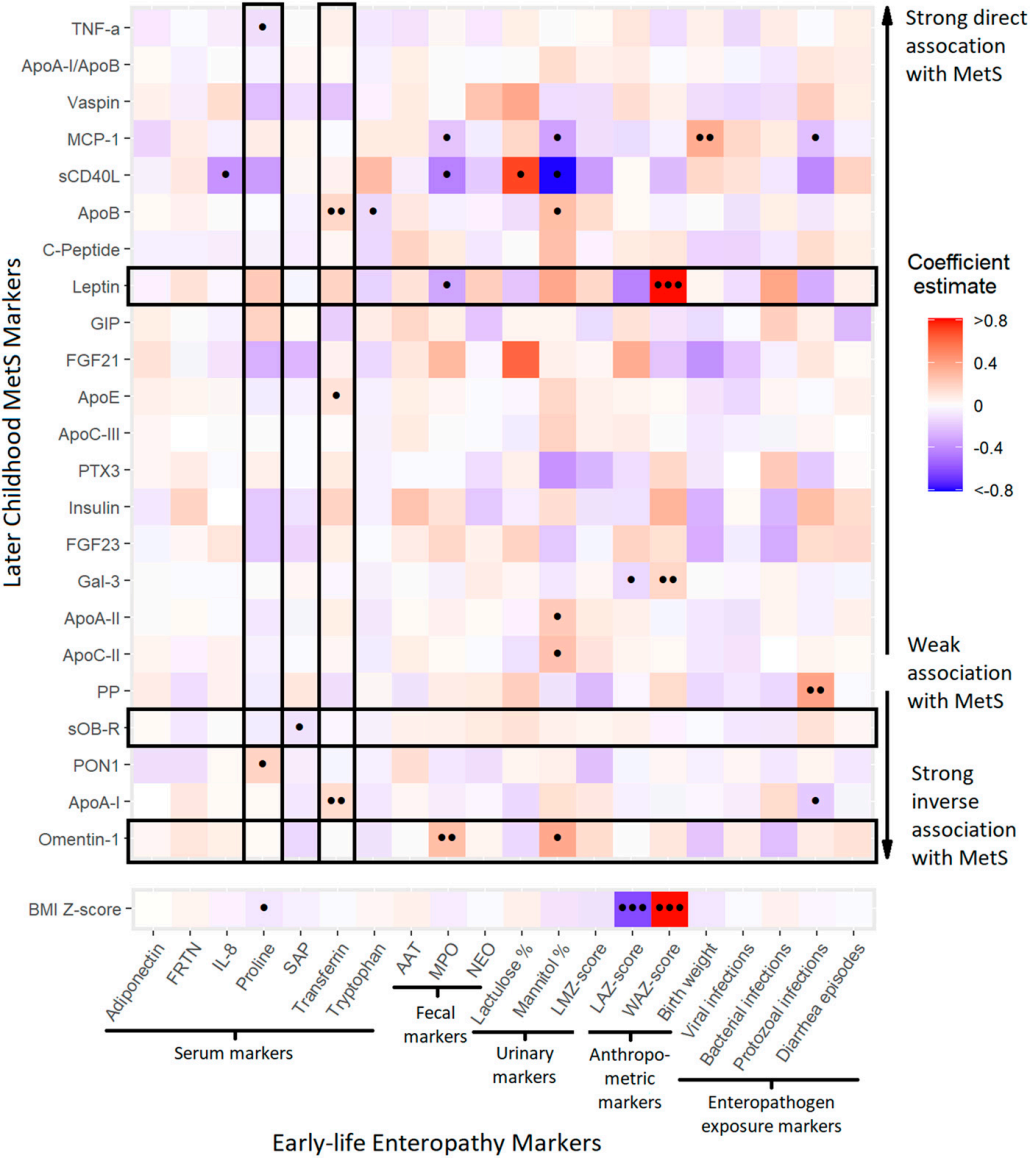
Heat map matrix of the coefficients from a multivariate regression model of the associations of multiple, standardized early-life (0–2 years) enteropathy markers with metabolic syndrome (MetS) biomarkers later in childhood (2–5 years), ordered by the strength and direction of their documented association with MetS, adjusting for gender, income, and age at follow-up. Unadjusted significance levels for individual coefficients are indicated with dots (•••*P *< 0.001, ••*P* = 0.001–0.01, •*P* = 0.01–0.05), and combinations of outcomes and exposures that were significant at the Bonferroni-adjusted α ≤ 0.05/*m* level (where *m* is either the number of outcomes or the number of early-life markers, depending on which is being jointly tested) according to the Wald test are outlined. AAT, alpha-1-antitrypsin; Apo, apolipoprotein; BMI, body mass index; FGF, fibroblast growth factor; FRTN, ferritin; Gal-3, galectin-3; GIP, gastric inhibitory polypeptide; IL, interleukin; LAZ, length-to-age z-score; LMZ, lactulose-to-mannitol ratio z-scores; MCP-1, monocyte chemoattractant protein 1; MPO, myeloperoxidase; NEO, neopterin; PON1, paraoxonase 1; PP, pancreatic polypeptide; PTX3, pentraxin 3; SAP, serum amyloid P-component; sCD40L, soluble CD40-ligand; sOB-R, soluble leptin receptor; TNF, tumor necrosis factor; Vaspin, visceral adipose tissue-derived serpin; WAZ, weight-to-age z-score. * BMI z-score is included as comparator clinical MetS outcome.

The heat map matrix in Figure 3 shows the coefficient estimates from a multivariate linear regression model of the associations between the set of standardized early-life markers and the cardiometabolic biomarker and comparator clinical outcomes in later childhood, adjusting for the covariates gender, income, exclusive breastfeeding, and maternal BMI. The serum biomarkers are ordered along the *y*-axis according to the strength and direction of their documented association with MetS based on a review of the literature, and results for BMI z-score are presented separately because these are from a single outcome, not the multivariate model. Multiple early-life exposure markers showed strong or statistically significant associations with the biomarker outcomes measured in later childhood. The largest and most statistically significant effect size observed in the multivariate model by some margin was the direct association of WAZ with leptin, whereas the similar association of urinary lactulose with sCD40L, and the corresponding inverse association between urinary mannitol with that same cytokine were also notable. Mannitol was also significantly associated directly with ApoC-II, ApoA-II, ApoB and omentin-1, and inversely with MCP-1; in each such case, the corresponding effect for lactulose was in the opposing direction. By contrast, for the lactulose-to-mannitol ratio z-scores (calculated from the two urinary biomarkers, but unavailable for 24-month samples because of the lack of standard reference values at that age), no strong or statistically significant effects were observed for any MetS biomarker. There was also a tendency for WAZ and LAZ to have opposite effects on the same biomarker in the adjusted model that were not apparent in the unadjusted correlation analysis. Three outcomes were statistically significant at the Bonferroni-adjusted α level across the entire set of exposures in the multivariate model—leptin, sOB-R, and omentin-1—the biomarkers with the strongest inverse association with MetS documented in the literature by our assessment. sCD40L was strongly inversely associated with IL-8, proline, MPO, and protozoal infections. Although MPO showed strong inverse associations with MCP-1 as well as sCD40L and leptin, and a direct effect on omentin-1, the marker did not retain statistical significance at the Bonferroni-adjusted α level across the entire set of MetS biomarkers. Proline and transferrin were the only early-life markers that met this criterion of correction for multiple comparisons, with the latter showing statistically significant direct associations with ApoA-I, ApoB, and ApoE. In the model of the single clinical comparator outcome BMI z-score, the strongest associations were with the two anthropometric metrics LAZ and WAZ. Associations with other exposures were smaller in magnitude than for many serum biomarker outcomes, although the association of proline was slightly statistically significant.

**Figure 4.**
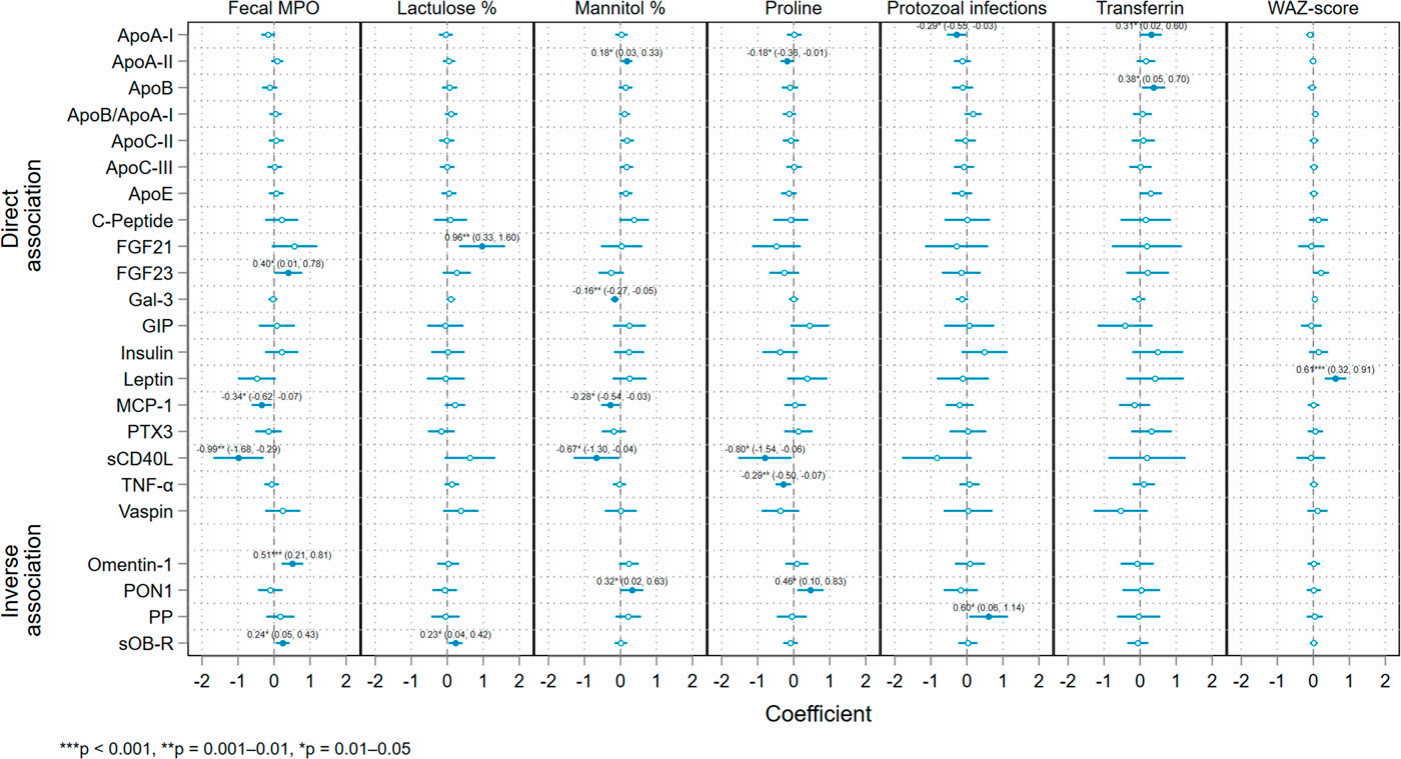
Coefficient estimates from regression models fitted for a subset of six early-life (0–2 years) exposures—myeloperoxidase (MPO), percent urinary lactulose and mannitol recovery, serum proline and transferrin, and protozoal infections (scaled so the effect is for an increase in 10 infections)—on each of the later-childhood (2–5 years) cardiometabolic biomarker outcomes adjusting for gender, income, and age with 95% CIs and significance levels. Outcomes are grouped by the documented direction of their associations with metabolic syndrome (direct or inverse). Apo, apolipoprotein; FGF, fibroblast growth factor; Gal-3, galectin-3; GIP, gastric inhibitory polypeptide; MCP-1, monocyte chemoattractant protein 1; PON1, paraoxonase 1; PP, pancreatic polypeptide; PTX3, pentraxin 3; sCD40L, soluble CD40-ligand; sOB-R, soluble leptin receptor; TNF, tumor necrosis factor; Vaspin, visceral adipose tissue-derived serpin; WAZ, weight-to-age z-score.

The subset of seven early-life markers selected for inclusion in the final regression models consisted of fecal MPO, percentage of urinary lactulose and mannitol recovery, serum proline and transferrin, WAZ, and total protozoal infections, with the latter rescaled so that the coefficients represent the effects of a 10-infection increment. Figure 4 is a plot of the coefficient estimates from the models fitted for the subset of early-life markers on each of the log_2_ MetS biomarker concentrations, adjusting for the covariates. As in the multivariate model, the effect of WAZ on leptin was large and highly statistically significant, with an increase of 1 z-score in average weight-for-age in early life associated with an almost two thirds increase (0.61; 95% CI, 0.32—0.91) in serum leptin concentrations later in childhood. However, this was the only outcome on which WAZ had a notable effect, and the largest identified absolute (unstandardized) effect size in the final models was of fecal MPO on sCD40L concentrations—a halving of the former in early life associated with a moderately statistically significant halving of the latter in later childhood (–0.99; 95% CI, –1.68 to –0.29). Effects on later sCD40L in the same direction and of comparable magnitude were observed for serum proline (–0.80; 95% CI, –1.54 to –0.06) and urinary mannitol (–0.67; 95% CI, –1.30 to –0.04), as well as for protozoal infections and, in the opposing direction, for lactulose, although in the latter two cases the CIs for the estimate narrowly spanned the null value.

The effect of early-life MPO on later omentin-1 levels increased in statistical significance in the final model compared with the multivariate model. An increase in omentin-1 concentrations of more than half (0.51; 95% CI, 0.21–0.81; *P* = 0.0009) was identified for every doubling in MPO levels, although none of the other six exposures in the final subset showed significant effects on omentin-1 (despite this having been one of only three outcomes in the multivariate model that was statistically significant at the Bonferroni-adjusted α level). MPO also showed large direct effects on FGF21 and FGF23, and a smaller effect on sOB-R, as well as indirect effects on sCD40L, leptin, and MCP-1. In addition to sCD40L, mannitol showed smaller magnitude inverse associations with MCP-1, galectin-3, and FGF23 (with the latter not significant at the α = 0.05 level), and small direct associations with ApoA-II, PON1, and C-peptide. For lactulose, significant direct associations were seen for sOB-R and FGF21, with the latter almost equivalent in magnitude to the large inverse MPO–sCD40L effect (0.96; 95% CI, 0.33–1.60). Serum proline concentration measured in early life demonstrated inverse associations with several later-life biomarkers that are directly associated with MetS—notably, sCD40L (–0.80; 95% CI, –1.54 to –0.06), TNF-α (–0.29; 95% CI, –0.50 to –0.07), ApoA-II (–0.18; 95% CI, –0.36 to –0.01]), insulin, FGF21, and Vaspin (with the latter three not significant at the α = 0.05 level), and a direct association with PON1 (0.56; 95% CI, 0.10—0.83), which is itself directly associated with MetS.

An increase in 10 total protozoal infections from birth to 2 years was associated with a 60% increase in PP (0.60; 95% CI, 0.06–1.14) and an almost one third decrease in ApoA-I (–0.29; 95% CI, –0.55 to –0.03), as well as a notable increase in insulin concentration and a decrease in sCD40L, although the latter two have CIs that narrowly included the null value. Although it was one of just two early-life markers to be statistically significant at the Bonferroni-adjusted α level in the initial multivariate regression, transferrin only showed associations with ApoB (0.38; 95% CI, 0.05–0.70) and ApoA-I (0.31; 95% CI, 0.02–0.6) that were significant at the at the α = 0.05 level in the final models, although large nonsignificant indirect effects on GIP and Vaspin were also observed.

## DISCUSSION

There is an urgent need to clarify the mechanisms that link enteric infections and EE in infancy with MetS later in life in a way that can inform intervention early in the disease process before significant end-organ damage and morbidity occur.^18^ The dissenting viewpoint espoused by Gustafson et al.^40^^,^^41^ that classifications of MetS in childhood and adolescence are instable and of limited relevance has been countered convincingly in the intervening years and it is now widely accepted that MetS severity scores and typologies derived from markers ascertained early in life can have considerable clinical and prognostic utility.^42^^–^^45^ It is plausible that the documented associations between diarrhea in early infancy, undernutrition in early childhood, and MetS later in life are partially mediated by EE. Further evidence for this hypothesis comes from the observations that MetS is highly prevalent even in low-resource settings with high enteropathogen burdens and an absence of lifestyle risk factors, and that obesity and liver steatosis are associated with concurrent intestinal permeability in adults.^16^^,^^46^ Hypothesized pathways by which EE in infancy may be linked to subsequent MetS in childhood and beyond include depressed apolipoprotein production causing dyslipidemia and inflammation resulting from bacterial or lipopolysaccharide translocation.^13^ However, the magnitude and relative clinical significance of these pathways have yet to be characterized. Our study is an initial attempt to investigate the associations between markers of early-life environmental exposures and adipokine, apolipoprotein, and cytokine profiles later in childhood. It is also the first to report the distributions of this large panel of biomarkers in a cohort of young children.

According to our conceptual framework (Figure 1), the earliest metabolic alterations may manifest as disruptions to cytokine profiles. Although many cytokines tested for in this cohort could not be included because too many values were outside the detectable range (e.g., interferon-γ and various interleukins), three that were included (MCP-1, sCD40L, and, to a lesser extent, TNF-α) demonstrated notable associations with numerous early-life enteropathy markers. In particular, concentrations of sCD40L—a proinflammatory cytokine shown to correlate positively with concurrent insulin resistance, BMI, and waist circumference^47^^,^^48^—was strongly associated with lactulose and mannitol in opposite directions but not with z-scores of the lactulose-to-mannitol ratio, the more commonly used way of expressing these markers as an indicator of intestinal absorption and permeability.^49^ The lactulose-to-mannitol ratio z-scores did not include data from 24 months as a result of the lack of available standard values at that age, which may contribute to the difference in effect compared with the two component biomarkers individually. As a larger disaccharide molecule, lactulose is absorbed in greater proportions when permeability is high, as a result of mucosal cell damage, whereas the monosaccharide mannitol is instead absorbed in direct proportion to the healthy mucosal absorptive area.^34^^,^^49^ Consequently, inflammatory bowel syndromes (such as Crohn’s disease) are associated with greater excretion of urinary lactulose, conditions characterized by flattening of the villi (e.g., Celiac disease) with lower mannitol,^50^ and both enteropathies are associated with elevated sCD40L levels.^51^^,^^52^ Our analysis also identified similar inverse associations between mannitol and both MCP-1 and (non-significantly) pentraxin 3, which, like sCD40L, are inflammatory markers themselves correlated with adverse MetS outcomes.^53^^,^^54^

Potential biomarkers of increased adiposity that were available for this analysis include the proinflammatory adipokines leptin, Vaspin, FGF21, and TNF-α (the latter also a cytokine), which are known to increase as a result of the interacting effect of central obesity with the other four MetS components (hyperglycemia, hypertriglyceridemia, dyslipidemia, and hypertension).^23^ The direct association between enteropathy markers and leptin in the multivariate analysis may constitute further evidence that previously documented links between morbidity in early childhood and MetS in adulthood may be mediated by increased adiposity with EE as its etiology.^14^ Specifically, EE may disrupt metabolic function through pathways other than increased intestinal permeability, such as increased adiposity as a result of elevated energy harvest caused by gut dysbiosis.^30^ However, the strong effect of both urinary lactulose and fecal MPO on FGF21 and FGF23, adipokines associated with increased intestinal permeability in mice and obesity in humans, suggests a complex interrelationship between these risk factors in the early pathogenesis of MetS.^55^^,^^56^

As biomarkers of lipid metabolism, we hypothesize that disruptions to apolipoproteins become apparent later in MetS pathogenesis than for cytokines and adipokines (Figure 1), accompanying later stage adverse metabolic adaptations.^57^^,^^58^ Nevertheless, some small-magnitude associations with early-life exposures were observed here even for apolipoproteins measured in childhood, although only for ApoA-I (directly with transferrin, inversely with protozoal infections), ApoA-II (directly with MPO and mannitol, inversely with proline), and ApoB (directly with transferrin) were these statistically significant in the final models. Notably, the ApoB-to-ApoA-I ratio, thought to be a better predictor of insulin resistance and MetS than any single lipid fractions,^58^ showed no associations with early-life markers, whereas for its two component analytes, several associations in the same direction were observed. These simultaneous positive associations with transferrin, which correlates directly with infant growth in this cohort,^27^ suggest that both ApoA-I and ApoB in children from EE-endemic settings may predict nutritional status, because loss of intestinal surface area is associated with decreased levels of both apolipoproteins.^13^ We hypothesized that disruptions to cardiometabolic profiles precede later outward clinical manifestations of MetS and may therefore be detectable earlier. The results from the BMI z-score model would appear to support this because, with the exception of two closely related anthropometric exposures, associations between early-life markers and this clinical comparator outcome tended to be smaller than for the serum biomarker outcomes.

Although these results appear consistent with the conceptual framework outlined in Figure 1, others appear to be at odds with prior hypotheses and findings. Fecal MPO—a lysosomal protein found in neutrophils that is used as a marker of oxidative stress induced by inflammatory bowel disease (IBD) and EE, and is predictive of subsequent growth faltering in young children^27^^,^^29^^,^^59^^,^^60^— had an inverse association with sCD40L that was larger and more statistically significant than that of mannitol, as did proline, another documented predictor of poor growth and oxidative damage.^27^^,^^61^ Previous findings have shown plasma sCD40L to correlate positively with concurrent levels of inflammatory chemokines,^62^ so it is unclear why IL-8 as well as oxidative stress markers (MPO and proline) would be associated negatively with later levels of the cytokine in our study. Furthermore, one of the most statistically significant associations found here was the direct effect of MPO on omentin-1, an anti-inflammatory adipocytokine known for being inversely correlated with weight loss, beneficial for insulin-sensitizing and vasodilation, and protective against metabolic and other disorders.^63^^,^^64^ However, it is now understood that omentin-1 is also an acute-phase, anti-inflammatory reactant, and therefore may increase during the initial stages of disease and as a response to inflammation.^64^ Omentin-1 also has a protective role against oxidative stress, such as is produced by MPO-induced cell injury,^65^ which may explain the positive association between these two markers in our analysis. Because oxidative stress is closely implicated in the pathogenesis of atherosclerosis in MetS, assessing both markers in combination may be clinically relevant and necessary for predicting adverse health outcomes. This is consistent with previous findings that intestinal regulatory T cells are activated and more abundant in children with active inflammatory bowel disease than healthy control subjects.^66^

Previous longitudinal studies have shown a link between poor early childhood growth and increased incidence of CVD^67^ and glucose intolerance^68^ later in life. The fact that significant associations were observed for biochemical enteropathy markers but not for the anthropometric indicators in this study (with the exception of the WAZ–leptin association) suggests that EE may impact later metabolic outcomes via mechanisms that operate independently of nutritional status and for which growth is a marker, rather than a mediator. In contrast to previously published findings,^10^ we did not find strong associations between diarrheal episodes in early childhood and later MetS markers, and of the three pathogen taxa assessed, only cumulative protozoal infections showed substantial effects on the later biomarkers, and mostly only in the multivariate model, such as increases in insulin, PP, and sOB-R, and decreases in leptin, MCP-1, sCD40L, and ApoA-I concentrations. sCD40L protects against protozoal infections through its interaction with CD40 promoting cell-mediated immunity,^69^ whereas leptin mediates resistance to *E. histolytica*,^70^ so it is possible that these infections are acting as a marker of chronic deficiency in these two cytokines that lasts through childhood. However, leptin is secreted by colonic epithelial cells during acute inflammation, and both micro- and macro-parasitic intestinal infections can disrupt its production,^71^^–^^73^ so a causative relationship is also plausible. The chemokine MCP-1 is involved in the intestinal inflammatory immune response against infection with various pathogens; in our study, we demonstrate an increase in enteric protozoal infections associated with lower serum MCP-1 for the first time in a human population (Figure 3).^74^ Although this apparent suppression of proinflammatory cytokines MCP-1 and sCD40L resulting from protozoal infections may at first sight seem surprising, previous studies have documented diminished inflammatory immune responses after repeated intestinal parasite infections, perhaps as an immune tolerance response to prevent further tissue damage.^75^ Such a downmodulation mechanism may also underlie the equally surprising inverse associations of IL-8, MPO, and proline with sCD40L. The direct association between cumulative protozoal infections and PP suggests that the loss of appetite that is a common symptom of these infections may be mediated, as opposed to compensated for, by the appetite-suppressing peptide.^76^ The direct association between protozoal infections and blood insulin levels appears to have no precedent in prior literature. A possible explanation for protozoal infections showing more associations with later outcomes than viruses and bacteria may be that they are more prone to longer, chronic infections, suggesting that cumulative duration of infection may be more important than the number of discrete infection episodes.

Our analysis was subject to several limitations. Numerous cytokines that circulate at very low concentrations were not able to be quantified in the cohort and several of these (amylin, glucagon, interferon-γ, IL-1B, IL-17A) have direct associations with MetS, which may undercut the argument for enteropathy as an etiology for MetS. Other biomarkers of documented relevance were also unavailable, such as the adiponectin-to-leptin ratio,^780^ as adiponectin was only measured in the early-life assessments, and leptin was measured at outcome ascertainment. Furthermore, as with any cohort study, the possibility that the subjects retained under surveillance differed systematically from those lost to follow-up is a potential source of bias that should be considered when interpreting these findings. The principal limitation, however, is the relatively short interval that elapsed between ascertainment of the exposures and outcomes. Although MetS is increasingly recognized as manifesting in childhood, the subjects in this study may have still been too young at the time of follow-up (≤ 5 years) for the precursors of the conditions to have become fully apparent. This may explain why many of the effect sizes identified here were small in magnitude; however, the fact that they were apparent at all and statistically significant even in mid-childhood is itself striking. We anticipate that these divergences in metabolic profiles will continue into adolescence and beyond, and will be accelerated by puberty and the accompanying increases in insulin, growth hormone, and insulin-like growth factor-I secretion; changes in body composition; and slowing of metabolism.^78^ This highlights the need for longer follow-up at multiple stages of the life course in this and other cohorts.

In conclusion, early-life enteric infection and enteropathy markers are associated with numerous changes in adipokine, apolipoprotein, and cytokine profiles later in childhood consistent with those of an adverse cardiometabolic disease risk profile in this Peruvian birth cohort. In particular, markers of intestinal permeability and inflammation measured in urine (lactulose, mannitol) and stool (MPO, protozoal infections) during infancy may predict disruptions to cytokine and adipocytokine production in later childhood that are precursors to MetS in adulthood. Chronic or recurring enteric infections, such as by protozoan pathogens, may be more important drivers of these changes than symptomatic diarrhea or growth faltering.

## Supplemental files


Supplemental materials
