# Supplementary material for: Enteropathy Markers in Early Life Were Associated with Adipokine, Apolipoprotein, and Cytokine Profiles Consistent with an Adverse Cardiometabolic Disease Risk Profile Later in Childhood in a Peruvian Birth Cohort
Source: Am J Trop Med Hyg. 2022 Sep 12;107(4):754–65. doi: 10.4269/ajtmh.21-1024 (PMC9651527; doi:10.4269/ajtmh.21-1024)
Supplement: Supplementary file 1 [file tpmd211024.SD1.pdf]

Early-life enteric infection and enteropathy markers are associated with changes in adipokine, apolipoprotein and cytokine profiles later in childhood consistent with those of an adverse cardiometabolic disease risk profile in a Peruvian birth cohort.

Supplementary materials:

Table S1: Summary statistics of candidate outcome biomarkers of metabolic syndrome (MetS) in the Peruvian birth cohort and their documented association with MetS components[1]

Analyte	Mean	Std. Dev.	Min.	Max.	Available values (%)	Associations with MetS components	Reference
**Amylin (total)**	24.2	34.0	0.9	394.4	98 (49.7)	TG+, IR+, BMI+	(1)
**Apolipoprotein A-I (ApoA-I)**	976,317.1	461,016.9	181,996.7	3,423,800.0	197 (100.0)	HDL+, TG-, IR-, WC-, BP-	(2)
**Apolipoprotein A-II (ApoA-II)**	371,037.8	145,386.6	73,415.9	1,268,400.0	197 (100.0)	WC+	(3)
**Apolipoprotein B (ApoB)**	1,505,892.5	713,726.4	236,557.0	4,335,500.0	197 (100.0)	HDL-, TG+, IR+, WC+, BP+	(2)
**ApoB/ApoA-I ratio**	179,908.5	83,269.4	30,553.7	503,804.5	197 (100.0)	HDL-, TG+, IR+, WC+, BP+	(2)
**Apolipoprotein C-II (ApoC-II)**	310,206.2	122,095.7	54,546.3	727,158.4	195 (99.0)	TG+	(4)
**Apolipoprotein C-III (ApoC-III)**	77,958.6	36,504.0	15,387.6	221,063.7	197 (100.0)	TG+, IR+, BP+, LDL+	(5)
**Apolipoprotein E (ApoE)**	820.4	646.6	19.9	2,683.4	190 (96.4)	TG+, IR+, BMI+	(6)
**C-Peptide**	324.8	498.3	1.2	3,008.8	183 (92.9)	HDL-, TG+, IR+, BMI+	(7)
**Fibroblast growth factor 21 (FGF21)**	268.1	386.9	2.0	2,121.7	196 (99.5)	HDL-, TG+, BMI+, WC+	(8)
**Fibroblast growth factor 23 (FGF23)**	21,491.4	6,626.1	11,402.2	46,082.4	177 (89.8)	HDL-, TG+, BMI+, WC+	(9)
**Galectin-3 (Gal-3)**	6.7	32.3	0.0	437.1	15 (7.6)	IR+, BMI+	(10)
**Ghrelin**	24.2	34.0	0.9	394.4	98 (49.7)	HDL+, IR-, BMI-, BP-	(11)

[1] +, direct association; -, inverse association; TG, triglycerides; IR, insulin resistance; BMI, body mass index/obesity; WC, waist circumference/central adiposity; BP, blood pressure; HDL, high-density lipoprotein cholesterol; LDL, low-density lipoprotein cholesterol. All analyte concentrations are in units of pg/mL, except for the apolipoproteins which are in ng/mL.

Table S1: Summary statistics of candidate outcome biomarkers of metabolic syndrome (MetS) in the Peruvian birth cohort and their documented association with MetS components[1]

Analyte	Mean	Std. Dev.	Min.	Max.	Available values (%)	Associations with MetS components	Reference
**Gastric Inhibitory Polypeptide (GIP)**	362.2	279.6	1.8	1,365.2	193 (98.0)	IR+, BMI+	(12)
**Glucagon-like Peptide 1 (GLP-1) Active/Total**	2.9	10.7	0.0	129.0	21 (10.7)	HDL+, TG-, IR-	(13)
**Glucagon**	35.8	27.9	2.5	207.3	84 (42.6)	IR+	(14)
**Interferon gamma (IFN-γ)**	1.4	3.8	0.0	23.9	17 (8.6)	IR+	(15)
**Interleukin 1 beta (IL-1β)**	0.2	0.7	0.0	4.4	9 (4.6)	IR+	(16)
**Interleukin 4 (IL-4)**	1.1	4.6	0.0	44.0	14 (7.1)	IR-, BMI-	(17)
**Interleukin 6 (IL-6)**	5.7	36.4	0.0	428.5	35 (17.8)	IR+, HDL-	(18)
**Interleukin 10 (IL-10)**	0.9	8.6	0.0	114.2	4 (2.0)	HDL+, TG-, IR-	(19)
**Interleukin 17A (IL-17A)**	0.1	1.0	0.0	8.0	3 (1.5)	IR+, BMI+	(20)
**Interleukin 22 (IL-22)**	2.8	9.0	0.0	57.0	16 (8.1)	IR-	(21)
**Interleukin 23 (IL-23)**	5.4	24.5	0.0	178.4	8 (4.1)	BMI-	(22)
**Interleukin 33 (IL-33)**	1.9	10.1	0.0	76.0	8 (4.1)	IR-, BMI-	(23)
**Insulin**	1.8	16.1	0.0	187.4	2 (1.0)	IR+, BP+	(24)
**Leptin**	148.9	215.7	0.3	1,187.4	115 (58.4)	IR+, BMI+, BP+	(25)
**Monocyte chemoattractant protein-1 (MCP-1)**	14.2	22.4	0.0	165.1	72 (36.5)	HDL-, TG+, IR+, BMI+, BP+	(26)
**Omentin-1**	431.0	460.1	27.5	2,613.2	132 (67.0)	IR-, BMI-, WC-	(27)

Table S1: Summary statistics of candidate outcome biomarkers of metabolic syndrome (MetS) in the Peruvian birth cohort and their documented association with MetS components[1]

Analyte	Mean	Std. Dev.	Min.	Max.	Available values (%)	Associations with MetS components	Reference
**Pentraxin 3 (PTX3)**	6,338.0	9,172.5	396.4	51,220.4	196 (99.5)	HDL-, TG+, IR+	(28)
**Paraoxonase-1 (PON1)**	5,113,437.7	3,143,770.4	9,927.5	21,358,020.0	190 (96.4)	HDL+, TG-, IR-, BMI-	(29)
**Pancreatic polypeptide (PP)**	348.2	281.6	11.7	1,261.1	176 (89.3)	BMI-, IR-	(30)
**Peptide tyrosine tyrosine (PYY)**	101.2	70.4	10.3	461.1	9 (4.6)	BMI-, IR-	(31)
**Soluble CD40-ligand (sCD40L)**	268.0	483.3	0.4	3,423.2	179 (90.9)	IR+, BMI+, WC+	(32)
**Soluble Leptin Receptor (sOB-R)**	35,967.8	14,642.5	7,396.1	78,243.8	197 (100.0)	HDL+, IR-, WC-	(33)
**Tumor Necrosis Factor Alpha (TNF-α)**	12.1	5.3	2.8	28.5	196 (99.5)	IR+, BMI+, WC+, TG+, BP+, HDL-	(34)
**Visceral adipose tissue-derived serpin (Vaspin)**	248.2	498.4	14.4	2,746.9	175 (88.8)	IR+, BMI+, TG+	(35)

Table S2: Definitions and distributions of early-life enteric infection and enteropathy markers in the Peruvian birth cohort		
**Variable**	**Definition**	**Mean (Std. Dev)**
**Exposures**		
Adiponectin	Within-subject average of plasma concentrations at 7, 15 and 24 months (ug/mL)	9.2 (2.7)
Ferritin (FRTN)	Within-subject average of plasma concentrations at 7, 15 and 24 months (ng/mL)	31.9 (27.3)
Interleukin-8 (IL-8)	Within-subject average of plasma concentrations at 7, 15 and 24 months (pg/mL)	12.7 (8.9)
Proline	Within-subject average of plasma concentrations at 7, 15 and 24 months (μM)	296.4 (90.2)
Serum Amyloid P-Component (SAP)	Within-subject average of plasma concentrations at 7, 15 and 24 months (ug/mL)	7.2 (2.0)
Serotransferrin (Transferrin)	Within-subject average of plasma concentrations at 7 and 15 months (mg/dl)	312.7 (72.8)
Tryptophan	Within-subject average of plasma concentrations at 7, 15 and 24 months (umol/L)	52.2 (9.3)
Alpha-1-Antitrypsin (AAT)	Within-subject average of fecal concentrations, 0-24 months (mg/g)	0.5 (0.2)
Myeloperoxidase (MPO)	Within-subject average of fecal concentrations, 0-24 months (mg/g)	10,905.9 (3662.9)
Neopterin (NEO)	Within-subject average of fecal concentrations, 0-24 months (nmol/L)	2,405.7 (706.7)
Lactulose %	Within-subject average of percent urinary lactulose recovery at 3, 6, 9, 15 and 24 months	0.3 (0.1)
Mannitol %	Within-subject average of percent urinary mannitol recovery at 3, 6, 9, 15 and 24 months	2.7 (1.3)
Lactulose:mannitol (LM) Z-score	Within-subject average of urinary LM ratio-for-age Z-score at 3, 6, 9 and 15 months	0.6 (0.4)
LAZ-score	Within-subject average of length-for-age Z-score, 0-24 months	-1.5 (0.8)
WAZ-score	Within-subject average of weight-for-age Z-score, 0-24 months	-0.6 (0.9)
Birth weight	Weight at birth (kg)	3.1 (0.4)
Viral infections	Within-subject total discrete enteric viral infections, 0-24 months	16.4 (5.6)
Bacterial infections	Within-subject total discrete enteric bacterial infections, 0-24 months	23.9 (6.6)
Protozoal infections	Within-subject total discrete enteric protozoal infections, 0-24 months	8.4 (3.6)
Diarrhea episodes	Within-subject total discrete diarrheal episodes, 0-24 months	9.1 (5.3)
**Covariates**		
Sex	Subject's sex	54.8% male
Household income	Average monthly income for subject's household (soles)	136.4 (52.9)
Exclusive breastfeeding	Proportion of days in which the subject was exclusively breastfed from age 0 – 6 months	0.46 (0.31)
Mother's BMI	Body mass index of the subjects' mother at the time of enrollment	13.0 (1.2)

Early-life enteric infection and enteropathy markers are associated with changes in adipokine, apolipoprotein and cytokine profiles later in childhood consistent with those of an adverse cardiometabolic disease risk profile in a Peruvian birth cohort.

References

1. Reinehr T, de Sousa G, Niklowitz P, Roth CL. Amylin and Its Relation to Insulin and Lipids in Obese Children Before and After Weight Loss*. Obesity [Internet]. 2007 Aug 1 [cited 2020 Dec 22];15(8):2006–11. Available from: http://doi.wiley.com/10.1038/oby.2007.239

2. Sniderman AD, Faraj M. Apolipoprotein B, apolipoprotein A-I, insulin resistance and the metabolic syndrome. Curr Opin Lipidol [Internet]. 2007 Dec [cited 2020 Dec 22];18(6):633–7. Available from: http://journals.lww.com/00041433-200712000-00005

3. van 't Hooft FM, Ruotolo G, Boquist S, de Faire U, Eggertsen G, Hamsten A. Human Evidence That the Apolipoprotein A-II Gene Is Implicated in Visceral Fat Accumulation and Metabolism of Triglyceride-Rich Lipoproteins. Circulation [Internet]. 2001 Sep 11 [cited 2020 Dec 22];104(11):1223–8. Available from: https://www.ahajournals.org/doi/10.1161/hc3601.095709

4. Kei AA, Filippatos TD, Tsimihodimos V, Elisaf MS. A review of the role of apolipoprotein C-II in lipoprotein metabolism and cardiovascular disease. Vol. 61, Metabolism: Clinical and Experimental. W.B. Saunders; 2012. p. 906–21.

5. Florez H, Mendez A, Casanova-Romero P, Larreal-Urdaneta C, Castillo-Florez S, Lee D, et al. Increased apolipoprotein C-III levels associated with insulin resistance contribute to dyslipidemia in normoglycemic and diabetic subjects from a triethnic population. Atherosclerosis. 2006 Sep 1;188(1):134–41.

6. Kypreos KE, Karagiannides I, Fotiadou EH, Karavia EA, Brinkmeier MS, Giakoumi SM, et al. Mechanisms of obesity and related pathologies: Role of apolipoprotein E in the development of obesity. FEBS J [Internet]. 2009 Oct 1 [cited 2020 Dec 22];276(20):5720–8. Available from: http://doi.wiley.com/10.1111/j.1742-4658.2009.07301.x

7. Banu S, R. Jabir N, N. Manjunath C, Shakil S, A. Kamal M. C-Peptide and its Correlation to Parameters of Insulin Resistance in the Metabolic Syndrome. CNS Neurol Disord - Drug Targets. 2012 Nov 1;10(8):921–7.

8. Zhang X, Yeung DCY, Karpisek M, Stejskal D, Zhou ZG, Liu F, et al. Serum FGF21 levels are increased in obesity and are independently associated with the metabolic syndrome in humans. Diabetes [Internet]. 2008 May 1 [cited 2020 Dec 22];57(5):1246–53. Available from: http://dx.doi.org/10.2337/db07-1476.

9. Mirza MAI, Alsiö J, Hammarstedt A, Erben RG, Michaëlsson K, Tivesten Å, et al. Circulating Fibroblast Growth Factor-23 Is Associated With Fat Mass and Dyslipidemia in Two Independent Cohorts of Elderly Individuals. 2010 [cited 2020 Dec 22]; Available from: http://atvb.ahajournals.org.

10. Pang J, Nguyen VT, Rhodes DH, Sullivan ME, Braunschweig C, Fantuzzi G. Relationship of galectin-3 with obesity, IL-6, and CRP in women. J Endocrinol Invest

[Internet]. 2016 Dec 1 [cited 2020 Dec 22];39(12):1435–43. Available from: https://pubmed.ncbi.nlm.nih.gov/27444618/

11. Purnell JQ, Weigle DS, Breen P, Cummings DE. Ghrelin Levels Correlate with Insulin Levels, Insulin Resistance, and High-Density Lipoprotein Cholesterol, But Not with Gender, Menopausal Status, or Cortisol Levels in Humans. J Clin Endocrinol Metab [Internet]. 2003 Dec 1 [cited 2020 Dec 22];88(12):5747–52. Available from: https://academic.oup.com/jcem/article-lookup/doi/10.1210/jc.2003-030513

12. Miyawaki K, Yamada Y, Ban N, Ihara Y, Tsukiyama K, Zhou H, et al. Inhibition of gastric inhibitory polypeptide signaling prevents obesity. Nat Med [Internet]. 2002 Jun 17 [cited 2020 Dec 22];8(7):738–42. Available from: http://medicine.nature.com

13. Meier JJ, Gethmann A, Götze O, Gallwitz B, Holst JJ, Schmidt WE, et al. Glucagon-like peptide 1 abolishes the postprandial rise in triglyceride concentrations and lowers levels of non-esterified fatty acids in humans. Diabetologia. 2006 Mar;49(3):452–8.

14. Færch K, Vistisen D, Pacini G, Torekov SS, Johansen NB, Witte DR, et al. Insulin resistance is accompanied by increased fasting glucagon and delayed glucagon suppression in individuals with normal and impaired glucose regulation. Diabetes [Internet]. 2016 Nov 1 [cited 2020 Dec 22];65(11):3473–81. Available from: http://www.diabetesjournals

15. O'Rourke RW, White AE, Metcalf MD, Winters BR, Diggs BS, Zhu X, et al. Systemic inflammation and insulin sensitivity in obese IFN-γ knockout mice. Metabolism. 2012 Aug 1;61(8):1152–61.

16. Jager J, Grémeaux T, Cormont M, Le Marchand-Brustel Y, Tanti J-F. Interleukin-1β-Induced Insulin Resistance in Adipocytes through Down-Regulation of Insulin Receptor Substrate-1 Expression. Endocrinology [Internet]. 2007 Jan 1 [cited 2020 Dec 22];148(1):241–51. Available from: https://academic.oup.com/endo/article/148/1/241/2501249

17. Tsao CH, Shiau MY, Chuang PH, Chang YH, Hwang J. Interleukin-4 regulates lipid metabolism by inhibiting adipogenesis and promoting lipolysis. J Lipid Res [Internet]. 2014 Mar 1 [cited 2020 Dec 22];55(3):385–97. Available from: http://www.jlr.org

18. Zuliani G, Volpato S, Blè A, Bandinelli S, Corsi AM, Lauretani F, et al. High interleukin-6 plasma levels are associated with low HDL-C levels in community-dwelling older adults: The InChianti study. Atherosclerosis. 2007 Jun 1;192(2):384–90.

19. Hong EG, Hwi JK, Cho YR, Kim HJ, Ma Z, Yu TY, et al. Interleukin-10 prevents diet-induced insulin resistance by attenuating macrophage and cytokine response in skeletal muscle. Diabetes [Internet]. 2009 Nov 1 [cited 2020 Dec 22];58(11):2525–35. Available from: http://diabetes.diabetesjournals.org

20. Fabbrini E, Cella M, McCartney SA, Fuchs A, Abumrad NA, Pietka TA, et al. Association between specific adipose tissue CD4+ T-cell populations and insulin resistance in obese individuals. Gastroenterology. 2013 Aug 1;145(2):366-374.e3.

Early-life enteric infection and enteropathy markers are associated with changes in adipokine, apolipoprotein and cytokine profiles later in childhood consistent with those of an adverse cardiometabolic disease risk profile in a Peruvian birth cohort.

21. Dalmas E, Donath MY. A role for interleukin-22 in the alleviation of metabolic syndrome [Internet]. Vol. 20, Nature Medicine. Nature Publishing Group; 2014 [cited 2020 Dec 22]. p. 1379–81. Available from: https://pubmed.ncbi.nlm.nih.gov/25362255/

22. Martins LMS, Perez MM, Pereira CA, Costa FRC, Dias MS, Tostes RC, et al. Interleukin-23 promotes intestinal T helper type17 immunity and ameliorates obesity-associated metabolic syndrome in a murine high-fat diet model. Immunology [Internet]. 2018 Aug 1 [cited 2020 Dec 22];154(4):624–36. Available from: http://doi.wiley.com/10.1111/imm.12946

23. Miller AM, Asquith DL, Hueber AJ, Anderson LA, Holmes WM, McKenzie AN, et al. Interleukin-33 induces protective effects in adipose tissue inflammation during obesity in mice. Circ Res [Internet]. 2010 Sep 3 [cited 2020 Dec 22];107(5):650–8. Available from: https://www.ahajournals.org/doi/10.1161/CIRCRESAHA.110.218867

24. Ferrannini E, Natali A, Capaldo B, Lehtovirta M, Jacob S, Yki-Järvinen for the European Grou H. Insulin Resistance, Hyperinsulinemia, and Blood Pressure. Hypertension [Internet]. 1997 Nov [cited 2020 Dec 22];30(5):1144–9. Available from: https://www.ahajournals.org/doi/10.1161/01.HYP.30.5.1144

25. Zhang Y, Scarpace PJ. The role of leptin in leptin resistance and obesity. Physiol Behav. 2006 Jun 30;88(3):249–56.

26. Deo R, Khera A, McGuire DK, Murphy SA, De P. Meo Neto J, Morrow DA, et al. Association among plasma levels of monocyte chemoattractant protein-1, traditional cardiovascular risk factors, and subclinical atherosclerosis. J Am Coll Cardiol. 2004 Nov 2;44(9):1812–8.

27. Tan BK, Adya R, Randeva HS. Omentin: A Novel Link Between Inflammation, Diabesity, and Cardiovascular Disease. Vol. 20, Trends in Cardiovascular Medicine. Elsevier; 2010. p. 143–8.

28. Zanetti M, Bosutti A, Ferreira C, Vinci P, Biolo G, Fonda M, et al. Circulating pentraxin 3 levels are higher in metabolic syndrome with subclinical atherosclerosis: Evidence for association with atherogenic lipid profile. Clin Exp Med [Internet]. 2009 Sep 24 [cited 2020 Dec 22];9(3):243–8. Available from: https://link.springer.com/article/10.1007/s10238-009-0039-z

29. Ferré N, Feliu A, García-Heredia A, Marsillach J, París N, Zaragoza-Jordana M, et al. Impaired paraoxonase-1 status in obese children. Relationships with insulin resistance and metabolic syndrome. Clin Biochem. 2013 Dec 1;46(18):1830–6.

30. Rabiee A, Galiatsatos P, Salas-Carrillo R, Thompson MJ, Andersen DK, Elahi D. Pancreatic polypeptide administration enhances insulin sensitivity and reduces the insnlin requirement of patients on insulin pump therapy. J Diabetes Sci Technol [Internet]. 2011 Nov 1 [cited 2020 Dec 22];5(6):1521–8. Available from: http://journals.sagepub.com/doi/10.1177/193229681100500629

Early-life enteric infection and enteropathy markers are associated with changes in adipokine, apolipoprotein and cytokine profiles later in childhood consistent with those of an adverse cardiometabolic disease risk profile in a Peruvian birth cohort.

31. Mittelman SD, Klier K, Braun S, Azen C, Geffner ME, Buchanan TA. Obese Adolescents Show Impaired Meal Responses of the Appetite-Regulating Hormones Ghrelin and PYY. Obesity [Internet]. 2010 May 1 [cited 2020 Dec 22];18(5):918–25. Available from: http://doi.wiley.com/10.1038/oby.2009.499

32. Unek IT, Bayraktar F, Solmaz D, Ellidokuz H, Sisman AR, Yuksel F, et al. The levels of soluble CD40 ligand and C-reactive protein in normal weight, overweight and obese people. Clin Med Res [Internet]. 2010 Jul 1 [cited 2020 Dec 22];8(2):89–95. Available from: http://www.clinmedres.org/content/8/2/89.full

33. Hamnvik OPR, Liu X, Petrou M, Gong H, Chamberland JP, Kim EH, et al. Soluble leptin receptor and leptin are associated with baseline adiposity and metabolic risk factors, and predict adiposity, metabolic syndrome, and glucose levels at 2-year follow-up: The Cyprus Metabolism Prospective Cohort Study. Metabolism. 2011 Jul 1;60(7):987–93.

34. Reinehr T, Roth CL. Inflammation Markers in Type 2 Diabetes and the Metabolic Syndrome in the Pediatric Population [Internet]. Vol. 18, Current Diabetes Reports. Current Medicine Group LLC 1; 2018 [cited 2020 Dec 22]. p. 1–12. Available from: https://doi.org/10.1007/s11892-018-1110-5

35. Youn BS, Klöting N, Kratzsch J, Lee N, Park JW, Song ES, et al. Serum vaspin concentrations in human obesity and type 2 diabetes. Diabetes [Internet]. 2008 Feb 1 [cited 2020 Dec 22];57(2):372–7. Available from: http://diabetes.diabetesjournals.orgon8novem-http//dx.
